# The application of shotgun metagenomics to the diagnosis of granulomatous amoebic encephalitis due to *Balamuthia mandrillaris*: a case report

**DOI:** 10.1186/s12883-021-02418-y

**Published:** 2021-10-09

**Authors:** Shota Hirakata, Yusuke Sakiyama, Akiko Yoshimura, Mei Ikeda, Katsunori Takahata, Yuichi Tashiro, Michiyoshi Yoshimura, Hitoshi Arata, Hajime Yonezawa, Mari Kirishima, Michiyo Higashi, Miho Hatanaka, Takuro Kanekura, Kenji Yagita, Eiji Matsuura, Hiroshi Takashima

**Affiliations:** 1 Department of Neurology and Geriatrics, Kagoshima University Graduate School of Medical and Dental Sciences, 8-35-1 Sakuragaoka, Kagoshima City, Kagoshima, 890-8520 Japan; 2 Department of Neurosurgery, Kagoshima University, 8-35-1 Sakuragaoka, Kagoshima City, Kagoshima, 890-8520 Japan; 3 Department of Pathology, Kagoshima University, 8-35-1 Sakuragaoka, Kagoshima City, Kagoshima, 890-8520 Japan; 4 Department of Dermatology, Kagoshima University, 8-35-1 Sakuragaoka, Kagoshima City, Kagoshima, 890-8520 Japan; 5 Department of Parasitology, National Institute of Infectious Diseases, 1-23-1, Toyama, Shinjuku-ku, Tokyo, 162-8640 Japan

**Keywords:** Granulomatous amoebic encephalitis, *Balamuthia mandrillaris* infection, Necrotizing encephalitis, Next-generation sequencer, Shotgun metagenomics

## Abstract

**Background:**

Granulomatous amoebic encephalitis (GAE) is an infrequent and fatal infectious disease worldwide. Antemortem diagnosis in this condition is very difficult because clinical manifestations and neuroimaging are nonspecific.

**Case presentation:**

A 60-year-old Japanese woman was admitted with a chief complaint of left homonymous hemianopsia. Brain-MRI showed extensive necrotizing lesions enhanced by gadolinium, in the right frontal lobe, right occipital lobe, and left parietal lobe. Epithelioid granulomas of unknown etiology were found in the biopsied brain specimens. Shotgun metagenomic sequencing using a next-generation sequencer detected DNA fragments of *Balamuthia mandrillaris* in the tissue specimens. The diagnosis of granulomatous amoebic encephalitis was confirmed using an amoeba-specific polymerase chain reaction and immunostaining on the biopsied tissues.

**Conclusions:**

Shotgun metagenomics is useful for the diagnosis of central nervous system infections such as GAE wherein the pathogens are difficult to identify.

**Supplementary Information:**

The online version contains supplementary material available at 10.1186/s12883-021-02418-y.

## Background

Granulomatous Amoebic Encephalitis (GAE), caused by the free-living amoeba *Balamuthia mandrillaris*, was first reported in humans in 1990 [1]. Currently, nearly 200 cases have been reported worldwide [2–4]. Clinical presentation and neuroimaging in this condition are nonspecific, and *B. mandrillaris* is rarely detected in common bacteriological tests for cerebrospinal fluid (CSF), making the diagnosis of GAE extremely difficult [2–5]. Herein, we report a case in which *B. mandrillaris* was identified by unbiased metagenomic sequencing of brain biopsy specimens and antemortem diagnosis of GAE was made.

## Case presentation

**Fig. 1:**
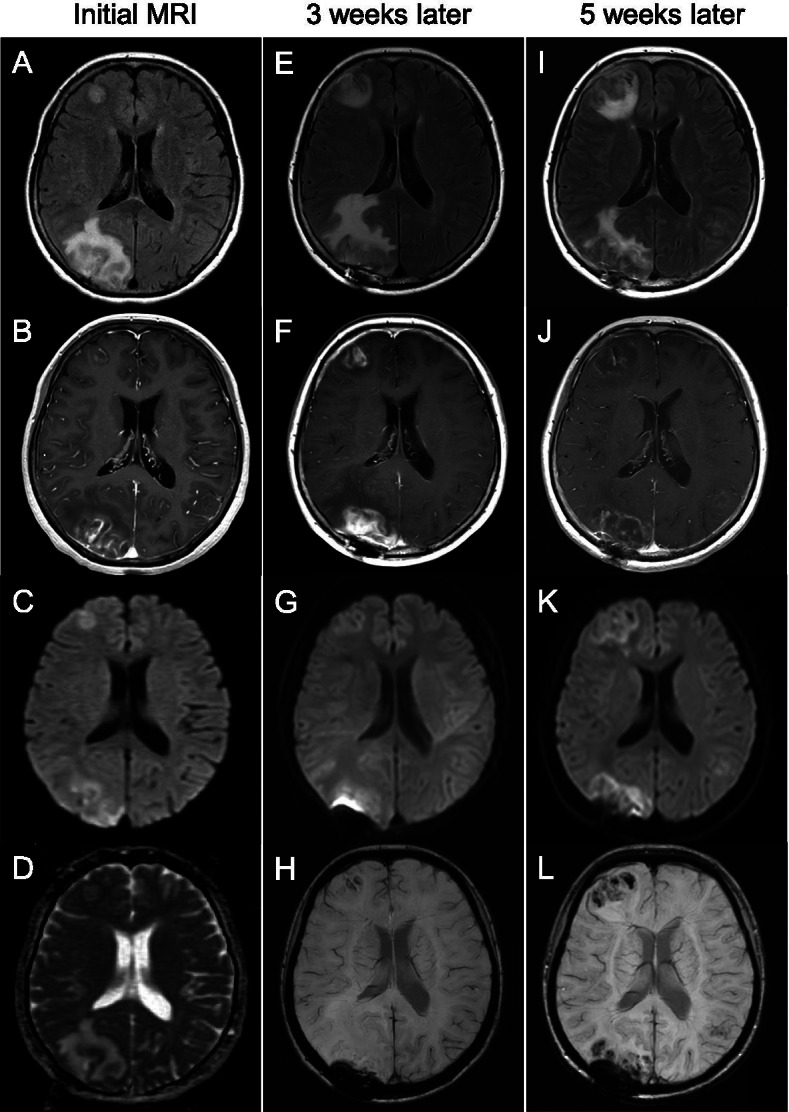
Neuroradiologic MRI findings. Magnetic resonance imaging (MRI) of the brain performed at the time of admission (**A–D**) and 3 weeks (**E–H**) and 5 weeks after (**I–L**) the initial MRI. Axial fluid-attenuated inversion recovery (FLAIR) **(A, E,** and **I)**, gadolinium-enhanced T1-weighted image (Gd-T1WI) (**B, F,** and **J**), diffusion-weighted image (DWI) **(C, G,** and **K)**, and susceptibility-weighted image (SWI) **(H** and **L)** showed multiple progressive nodules with hemorrhagic and necrotic lesions in the right frontal lobe, right occipital lobe, and left parietal lobe (not shown). Apparent diffusion coefficient (ADC) map on admission showed low signal intensity in the center areas and high signal intensity in the surrounding areas in the right occipital lobe lesion (**D**). The biopsied scar was found in the right occipital lobe **(E–L)**

A 60-year-old Japanese woman was admitted to our hospital because of visual field loss and unsteady gait. The patient spent time gardening as a hobby and was on infliximab and methotrexate therapy for rheumatoid arthritis. Two months before admission, her left visual field loss had steadily progressed, and she felt low-grade fever and general malaise. A high signal in the right occipital lobe and the left parietal lobe was detected in the MRI fluid-attenuated inversion recovery (FLAIR), and the lesions were enhanced by gadolinium (Fig. 1A–D). Brain biopsy of the right occipital lobe lesion demonstrated granulomatous inflammation, but no pathogenic micro-organisms were detected using either microscopic examination or culture. A diagnosis of neurosarcoidosis was made by the neurologists based on the brain biopsy findings.

The symptom of unsteady gait was temporarily relieved by intravenous methylprednisolone (IVMP); although, it worsened soon after discharge, followed by readmission to our department. At the time of readmission, there were no notable findings in the chest and abdomen and no uveitis was noted. A brown spot with a scab, about 10 millimeters in diameter, was observed inside the left upper arm. Neurologically, left lower visual field and mild weakness in the distal muscles of the upper limbs were observed. Deep tendon reflexes and muscle tonus in the upper and lower limbs were normal, and there was no ataxia or sensory impairment. Analysis of blood samples showed no abnormal findings in the blood cell count or the coagulation system. Serum angiotensin-converting enzyme II activity was 12.9 U/L and lysozyme level was 6.2 μg/mL. Antinuclear and antineutrophil cytoplasmic antibodies were negative. Specific antibodies for aquaporin 4 and myelin oligodendroglia were also negative. Tumor markers were not elevated. Antiparasitic antibodies and blood cultures were also negative. The CSF collected 1 week after brain biopsy was colorless and transparent, and the initial cerebrospinal pressure was 12 cmH_2_O. The CSF cell count was measured to be elevated at 43/μL (neutrophil 0/μL, lymphocyte 38 /μL, and monocyte 5/μL), in addition to the increased protein levels at 77.8 mg/dL. Cerebrospinal fluid sugar level was 86 mg/dL, while the blood glucose level was detected at 192 mg/dL. The CSF/serum ratio of albumin (Q-albumin) was elevated at 14.2 (CSF albumin: 57 mg/dL, serum albumin: 4010 mg/dL). Gram staining did not detect any bacteria, and common bacteria and acid-fast bacteria were not cultured from the CSF. *Cryptococcus neoformans* antigen, *Aspergillus* antigen, *Toxoplasma* antibody, and Human Polyomavirus 2 DNA were all negative. Transthoracic echocardiography showed no obvious thrombi or warts. Skin biopsy performed on the brown spot on the left upper arm showed dense lymphoplasmacytic inflammation distributing from the upper dermis to the subcutaneous adipose tissue with a few epithelioid cell granulomas. Contrast-enhanced computed tomography (CT) of the region from the cervix through the pelvis revealed no neoplastic lesions or bilateral hilar lymphadenopathy, and gallium scintigraphy did not reveal any abnormal accumulation.

Brain MRI after IVMP showed enlarged lesions with internal necrosis (Fig. 1E–H). At 16 days after admission, ceftriaxone (2 g/day), acyclovir (1.2 g/day), fosfluconazole (0.4 g/day) sulfamethoxazole (1.2 g/day), and trimethoprim (0.24 g/day) were initiated along with the second IVMP to cover both autoimmune encephalitis and infectious meningitis caused by bacteria, herpes virus, *Cryptococcus neoformans*, and parasites. However, the treatment response was poor, and new lesions appeared in the medulla oblongata (data not shown). Because the patient suddenly fell into a coma on day 21 after admission, intratracheal intubation was performed and ventilator management was started. Brain MRI revealed progressed necrosis and edema (Fig. 1I–L). A second brain biopsy with needle aspiration from reddish-brown tissue revealed necrotic tissue which was considered to be reflecting brain abscess, but no pathogenic micro-organisms were detected using either microscopic examination or culture. Furthermore, neither tumor nor granulomas were observed in the specimen.

**Fig. 2:**
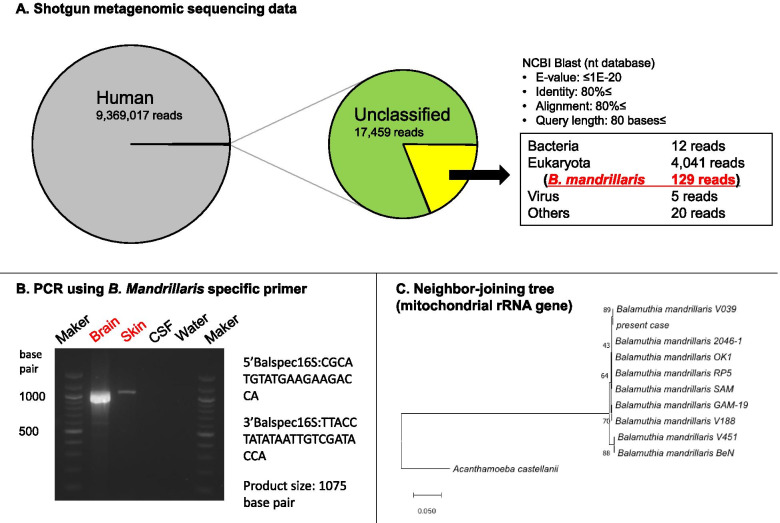
Result of metagenomic NGS, PCR and phylogenetic analysis. **A**, Analysis of metagenomic next-generation sequencing (NGS) results from a patient. The total reads distribution is shown on the left and the distribution of reads without the human genome is shown on the right. Genomic DNA was extracted from the biopsied brain samples using an extraction kit (QIAGEN, Tokyo, Japan). Shotgun metagenomic sequencing was performed using the MiSeq® NGS system (NextraXT prep kit®, Illumina) and a paired-end sequencing approach. For the identification of non-human DNA, the CLC Genomics Workbench was first used to query a human genome database (UCSC: hg38) and a human mRNA database (RefSeq release 54) and eliminate most human sequences. The remaining unmapped reads were subsequently analyzed by Nucleotide BLAST against the nt database from NCBI. A total of 129 reads showed a striking homology to *B. mandrillaris* under conditions of E-value <1e^− 20^, hit length > 80, and identity > 80%. **B**, Polymerase chain reaction analysis using species-specific primers revealed a positive reaction for *B. mandrillaris* in the brain and skin samples from the patient and a negative reaction for *B. mandrillaris* in the CSF from the patient and the water control. *C*, Phylogenetic analysis of *B. mandrillaris* mitochondrial small subunit rRNA gene was performed based on the neighbor-joining method using MEGA X software. The percentage trees in which the associated taxa cluster together in the bootstrap test (1000 replicates) are shown next to the branches. Scale bars indicate the genetic distance

**Fig. 3:**
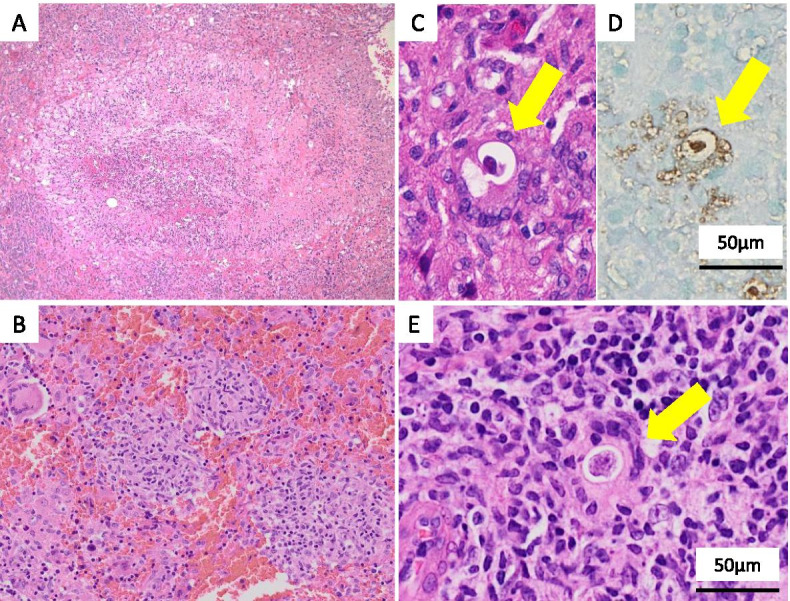
Pathological findings of brain and skin biopsy samples. Histopathological findings of the brain biopsy (**A**, **B**, **C** and **D**), and the skin biopsy (**E**). **A**, hematoxylin and eosin (H&E) stained section showing necrotic tissue surrounded by palisading epithelioid histiocytes, forming epithelioid granuloma with necrosis (original magnification × 100). **B**, Multiple epithelioid granulomas without necrosis were also seen (H&E stain, original magnification × 200). **C** and **D**, Multinucleated giant cell containing an amoebic trophozoite (**C**, **H&E** stain, highlighted by yellow arrow) was detected, which was confirmed by immunohistochemical staining with anti-*B. mandrillaris* rabbit antibody (**D**, highlighted by yellow arrow) (original magnification × 400). **E**, Skin biopsy also showed multinucleated giant cells containing amoebic trophozoite (H&E stain, highlighted by yellow arrow, original magnification × 400)

In order to identify the causative pathogenic micro-organism, the initial brain biopsy specimens were subjected to shotgun metagenomic sequencing using a next-generation sequencer (NGS). The DNA extracted from the brain specimens was comprehensively analyzed using Illumina sequencer (MiSeq®) and compared with the sequences registered in the database through the BLASTN analysis. As a result, 129 DNA fragments homologous to *B. mandrillaris* were identified from the 9 million DNA fragments derived from the brain specimens (Fig. 2A). The PCR analysis for the mitochondrial rRNA of *B. mandrillaris* [6] revealed bands of the PCR product in the patient’s brain and skin biopsy specimens (Fig. 2B, Additional file 1: Fig. S1). The pathogen detected from the present case was presumed to be a strain with a high degree of similarity to *B. mandrillaris* V039 based on phylogenetic analysis using MEGA X [7] (Fig. 2C). Based on these findings, histopathological specimens of the brain tissue (Fig. 3A***–***D) and skin biopsy (Fig. 3E) were re-evaluated microscopically. Brain biopsy of the right occipital lobe lesion demonstrated granulomatous inflammation, containing epithelioid granuloma with palisading necrosis (Fig. 3A) and multiple epithelioid granulomas without necrosis (Fig. 3B). Moreover, multinucleated giant cells containing amoebic trophozoites were seen on hematoxylin and eosin (H&E) stain (Fig. 3C) and these trophozoites were confirmed immunohistochemically with anti-*B. mandrillaris* rabbit antibody (Fig. 3D). Skin biopsy also showed a few amoebic trophozoites (Fig. 3E) which were confirmed by immunohistochemical staining. Thus, the patient was diagnosed with GAE caused by *B. mandrillaris*. The treatments with azithromycin (0.5 g/day), sulfamethoxazole (1.2 g/day), trimethoprim (0.24 g/day), and fosfluconazole (0.2 g/day) was initiated; however, the patient died 44 days after admission.

## Discussion and conclusion

It is extremely difficult to make a clinical diagnosis of GAE while the patient is still alive. Of the 18 cases of GAE in Japan including the present case, 16 cases were diagnosed at autopsy, and only one case was successfully treated [3, 8]. The reason for the difficulty in GAE diagnosis is the rapid disease progression and a lack of specific clinical symptoms [2, 5]. Moreover, the trophozoites and cysts of *B. mandrillaris* are difficult to detect in CSF [2–5] and rarely grow on agar plates inoculated with mammalian cell cultures used to isolate *B. mandrillaris* [1, 2]. Brain lesions associated with GAE can be visualized as occupied lesions with hemorrhage and necrosis on brain CT and MRI, although these are not disease-specific findings [9, 10]. Elevated antibody titers to *B. mandrillaris* are useful for diagnosis; however, these antibody tests are not commercially available. In addition, if the clinician does not suspect GAE, there is no opportunity to evaluate antibody titers. For these reasons, a biopsy of brain lesions is crucial for the diagnosis of GAE. Similar to the findings from brain imaging, the lesion exhibits hemorrhagic necrosis, chronic inflammatory infiltrates with sparse multinucleated giant cells, and diffuse lymphocytes and plasma cells [1]. Even if some amoeba trophozoites and cysts are present in the lesion, their identification may be challenging due to a general lack of familiarity with their morphology [11]. A background of extreme inflammation and necrosis can also risk masking the presence of the amoeba on routine histopathologic examination, such as in the present case.

The shotgun metagenomic sequencing using NGS has been demonstrated to be useful in the diagnosis of CNS infections that are difficult to diagnose [12–16]. Infectious and non-infectious CNS diseases are difficult to distinguish based on symptomology alone, and effective tests for rare pathogenic micro-organisms have not been established [12, 14]. Conventional diagnostic methods for CNS infections require several tests, including smear, culture, nucleic acid amplification, and serological tests, as well as a large number of CSF samples [16]. The shotgun metagenomic sequencing is an innovative diagnostic tool because it can facilitate a comprehensive evaluation of a variety of pathogenic microorganisms in a small amount of specimen using only a single assay [17]. In a study evaluating the efficacy of NGS for CNS infections of unknown etiology, 7 out of 204 cases were identified by metagenomic sequencing alone, contributing to the selection of effective treatments [14]. If this present case had undergone shotgun metagenomic sequencing at the time of the first brain biopsy, the patient could have been given more adequate treatment against the amoebae.

In conclusion, we made a diagnosis of GAE due to *B. mandrillaris* infection using shotgun metagenomic sequencing of biopsied brain specimen while the patient was still alive. Early diagnosis of amoebic encephalitis remains a challenge, and several cases may escape detection. We recommend that unbiased metagenomic sequencing of the affected tissues be performed in patients with CNS infections that are difficult to diagnose and treat, including amoebic encephalitis.

## Supplementary Information

**Additional file 1: Supplementary Figure S1.** Unprocessed original gel/blot images of polymerase chain reaction analysis using species-specific primers for *B. mandrillaris*.

## Data Availability

Not applicable.

## Abbreviations

GAE: Granulomatous amoebic encephalitis
CSF: Cerebrospinal fluid
MRI: Magnetic resonance imaging
IVMP: Intravenous methylprednisolone
NGS: Next-generation sequencer
