# Supplementary figures and images for: The application of shotgun metagenomics to the diagnosis of granulomatous amoebic encephalitis due to Balamuthia mandrillaris: a case report
Source: BMC Neurol. 2021 Oct 9;21:392. doi: 10.1186/s12883-021-02418-y (PMC8501540; doi:10.1186/s12883-021-02418-y)

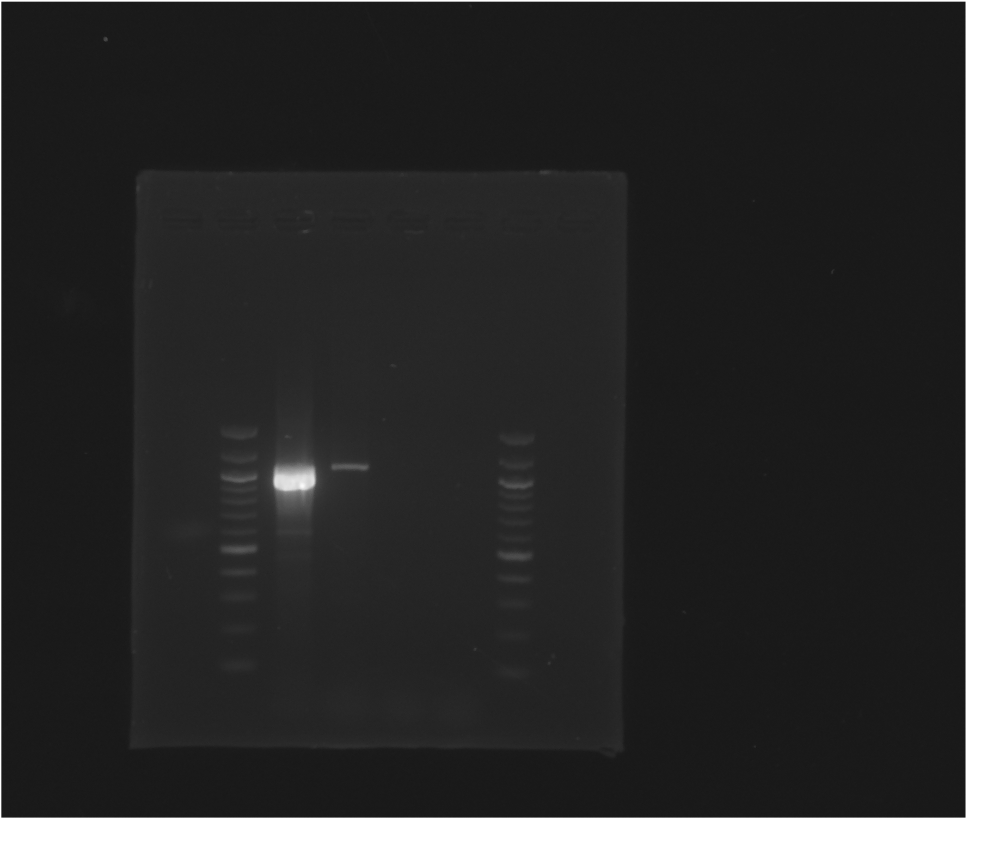

Supplement: Supplementary file 1 — Additional file 1: Supplementary Figure S1. Unprocessed original gel/blot images of polymerase chain reaction analysis using species-specific primers for B. mandrillaris. [file 12883_2021_2418_MOESM1_ESM.png]
